# Antioxidant and Anti-Inflammatory Activities of Endemic Plants of the Australian Wet Tropics

**DOI:** 10.3390/plants11192519

**Published:** 2022-09-26

**Authors:** Karma Yeshi, Roland Ruscher, Kim Miles, Darren Crayn, Michael Liddell, Phurpa Wangchuk

**Affiliations:** 1 Centre for Molecular Therapeutics, Australian Institute of Tropical Health and Medicine, James Cook University, Building E4 and E5, McGregor Rd, Smithfield, QLD 4878, Australia; 2 Australian Tropical Herbarium, James Cook University, Building E2, McGregor Rd, Smithfield, QLD 4878, Australia; 3 College of Science and Engineering, Centre for Tropical Environmental and Sustainability Science, Building E1, McGregor Rd, Smithfield, QLD 4878, Australia

**Keywords:** antioxidant, anti-inflammatory, mountaintop plants, phenolics, flavonoid, DPPH, reducing power

## Abstract

Plants have been a vital source of natural antioxidants since ancient times. Plants growing under various abiotic stress conditions often produce more defensive secondary metabolites such as phenolics, flavonoids, and terpenoids during adaptation to the environment. Many of these secondary metabolites are known to possess antioxidant and anti-inflammatory properties. This study tested seven plants sourced from the mountaintop areas (above 1000 m elevation) of Mount Lewis National Park (falls under the Wet Tropics of Queensland), Australia, for their antioxidant and anti-inflammatory activities. Of the seven studied plants, hydroethanolic extracts of six plants (*Leptospermum wooroonooran*, *Ceratopetalum hylandii*, *Linospadix apetiolatus*, *Garcinia brassii*, *Litsea granitica*, and *Polyscias willmottii*) showed high 2,2-diphenyl-1-picrylhydrazyl (DPPH)-free radical scavenging activity in a dose-dependent (25–1000 μg/mL) manner. At the highest concentration of 1 mg/mL, the DPPH free radical scavenged percentage varied between 75.4% and 92.3%. Only the species *Alyxia orophila* was inactive in the DPPH free radical scavenging assay. Pseudo-IC_50_ values of the extracts’ ferric reducing antioxidant power (FRAP) based on dose-response curves showed a significant positive correlation with total phenolic content. Five out of the seven plants, namely *G. brassii*, *C. hylandii*, *L. apetiolatus*, *L. wooroonooran*, and *A. orophila*, showed inhibitory effects on the secretion of proinflammatory cytokines, tumour necrosis factor (TNF), and interleukins (IL)-23 in a lipopolysaccharide (LPS)-stimulated human peripheral blood mononuclear cells (PBMCs) assay. The results of this study demonstrate the value of tropical mountaintop plants in the biodiscovery of antioxidant and anti-inflammatory lead compounds.

## 1. Introduction

The human body produces endogenously reactive oxygen species (ROS) and reactive nitrogen species (RNS), which serve as cell signalling molecules for normal biological functions and processes. However, the excessive formation of ROS can cause oxidative stress and disrupt normal physiological processes by damaging the antioxidant defences that protect the cells. Oxidative stress occurs when the body’s biological system cannot detoxify excessively accumulated ROS in cells [1,2]. The most common ROS species in disease states include hypochlorite, hydrogen peroxide, and a range of radical species, including the superoxide anion, singlet oxygen, hydroxyl, hydroperoxyl, peroxy, and alkoxy radicals [3]. The most common RNS are nitrogen dioxide, nitric oxide, and peroxynitrite radicals [4,5]. Under oxidative stress, an elevated level of reactive free radicals can damage vital cellular macromolecules such as nucleic acids, lipids, and proteins [6]. As a consequence, this imbalance in favour of ROS damages cells and tissues and, in many cases, leads to inflammation [7]. Both oxidative stress and inflammation contribute to the pathogenesis of many chronic diseases and metabolic disorders such as cancer, diabetes mellitus, rheumatoid arthritis, and neurodegenerative disorders such as Parkinson’s disease [6]. When the endogenous antioxidant defence mechanism becomes inadequate in the human body, antioxidant supplements often help reverse cell death and damage. Butylated hydroxyanisole and butylated hydroxytoluene are commonly available synthetic antioxidant supplements, but sustained use of these compounds is associated with carcinogenesis and hepatic damage [8].

Plants and their products, including fruits, vegetables, cereals, and herbs, are important dietary sources of natural antioxidants, such as polyphenols [9]. Polyphenols are chemical compounds characterised by the presence of multiple phenolic rings in their structures and can be categorised into four different classes, namely phenolic acids, flavonoids, stilbenes, and lignans [10]. Dietary consumption of plants rich in polyphenols and their products over a long period is known to confer protection against cardiac diseases [11,12], cancers [13,14], diabetes [15,16], osteoporosis [17], and neurodegenerative disorders [18,19]. Polyphenols are also used in the cosmetic industry as effective anti-aging agents [20]. So far, more than 8000 phenolic compounds have been reported, out of which more than half are identified as flavonoids [10].

Plants accumulate antioxidative and other protective compounds as a result of acclimation to various abiotic stress conditions, including high atmospheric ozone (O_3_) levels, drought, temperature, and ultraviolet (UV-B) radiation [21,22,23,24]. After exposure to these atmospheric conditions, plants develop the capacity to reprogram their physiology through either genetic or phenotypic responses, creating the novel gene products required to establish secondary metabolite biosynthetic pathways [25]. For example, in plants exposed to high atmospheric ozone (O_3_) levels, activation of the enzyme phenylalanine ammonia-lyase (PAL) triggers the phenylpropanoid pathway to produce defensive and cell wall fortifying compounds, such as phenolic acids, flavonoids, and monolignoids [26,27,28]. Glutathione, γ-aminobutyric acid (GABA), terpenoids, isopropanoids, and volatile organic compounds (VOC) are also produced in response to elevated O_3_ [29,30,31]. While drought stress increases the levels of phenolic acids (as demonstrated by *Camelia sinensis*) [23], plants exposed to high temperatures produce more terpenoids [32]. Similarly, exposure to UV-B radiation induces tree foliage to produce more phenolic acids and flavonoids as protective pigments [33,34].

Of all the plant species that grow in various ecological habitats, mountaintop plants are particularly susceptible to shifts in temperature and precipitation patterns [35,36]. A recent review by Yeshi et al. (2022) reported that mountaintop plants growing under elevated or high atmospheric conditions (e.g., CO_2_ and O_3_) tend to produce more polyphenols and terpenoids compared to plants at lower elevations [37]. This review also highlighted that the plant secondary metabolites (phenolic acids and flavonoids) generally possess better antioxidant and anti-inflammatory properties than other groups of secondary metabolites [38,39,40]. Based on an ecologically directed biorational approach, this study evaluated seven plant species endemic to mountaintop areas of the Wet Tropics of Queensland, Australia, for their major classes of phytochemicals and antioxidant and anti-inflammatory activities.

## 2. Results

### 2.1. Crude Extraction and Extract Yield

When 25 g of dried aerial (mostly leaves) parts of each plant were powdered and extracted with 150 mL of 80% ethanol (three times), *L. wooroonooran* yielded the greatest crude extract upon drying (16.8%), followed by *P. willmottii*, *L. granitica*, and *A. orophila*. *Ceratopetalum hylandii* had the lowest extract yield of only 3.5% (Table 1).

### 2.2. Major Classes of Phytochemical Analysis

Hydroethanolic extracts from seven species were screened for the presence of seven different classes of phytochemicals using the methods described by Wangchuk et al. [41]. The results are shown in Table 2. The extracts of *Garcinia brassii*, *C. hylandii*, and *A. orophila* showed positive tests for five major classes of phytochemicals; *L. granitica* and *P. willmottii* contained four classes of phytochemicals; and *L. wooroonooran* and *L. apetiolatus* contained three classes of phytochemicals. *Ceratopetalum hylandii* showed the presence of a high concentration of alkaloids, tannins, terpenoids, and cardiac glycosides. Extracts of all seven species showed the presence of tannins; all except *L. apetiolatus* tested positive for steroids; and all except *L. apetiolatus* and *G. brassii* tested positive for terpenoids. Anthraquinone glycosides were detected in *G. brassii* only.

### 2.3. Phenolic and Flavonoid Content

**Figure 1:**
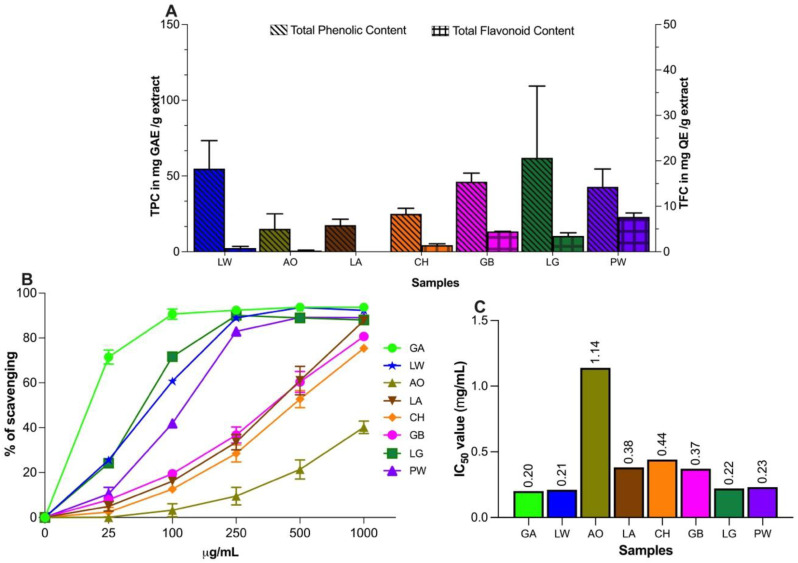
Phytochemical content and antioxidant activities of seven Wet Tropics plant extracts. (**A**) Phenolic and flavonoid content. (**B**) DPPH free radical scavenging activity at various concentrations compared with standard gallic acid. (**C**) The IC_50_ value (mg/mL) of DPPH free radical scavenging activity. All values are expressed as mean ± SD from two separate experiments (n = 3). GA—gallic acid (reference compound); LW—*Leptospermum wooroonooran*; AO—*Alyxia orophila*; LA—*Linospadix apetiolatus*; CH—*Ceratopetalum hylandii*; GB—*Garcinia brassii*; LG—*Litsea granitica*; PW—*Polyscias willmottii*.

Total phenolic content (TPC) was analysed for all seven plant species using Folin–Ciocalteu reagent, and the results were expressed as mg gallic acid equivalent (GAE)/g extract. Figure 1A summarises the results. *Litsea granitica* had the highest phenolic content (61.95 ± 0.09 mg GAE/g extract), followed by *L. wooroonooran* (54.89 ± 0.05 mg GAE/g extract) and *G. brassii* (46.21 ± 0.08 mg GAE/g extract). *Alyxia orophila* had the lowest phenolic content: 15.13 ± 0.02 mg GAE/g extract. Ranked phenolic content of all species was LG > LW > GB > PW > CH > LA > AO.

Total Flavonoid Content (TPC) in the seven species was expressed as mg of quercetin equivalent (QE)/g extract. The flavonoid content was comparatively lower than phenolic content in all plants. *Polyscias willmottii* showed the highest flavonoid content (22.98 ± 0.03), followed by *G. brassii* (13.38 ± 0.02) and *L. granitica* (10.41 ± 0.02), while *L. apetiolatus* and *A. orophila* had negligible flavonoid content (<1 mg QE/g extract) (Figure 1A). Ranked flavonoid content was PW > GB > LG > CH > LW > AO > LA.

### 2.4. DPPH Free Radical Scavenging Activity

The free radical scavenging activity of extracts from the seven species were measured in vitro by a DPPH assay [42,43]; 2,2-diphenyl-1-picrylhydrazyl (DPPH) is a stable radical used for quantifying antioxidant content. Figure 1B shows the dose-response scavenging effects of all seven species on the DPPH free radical at five different concentrations between 25 and 1000 μg/mL. Gallic acid was used as a reference compound. The scavenging effect for each species increased as their concentration increased. The percentage of DPPH scavenging effect (at the highest concentration of 1000 μg/mL) was highest in *L. wooroonooran* (92.3%, almost equivalent to the standard gallic acid = 93.7%), followed by *P. willmottii* (89.0%) and *L. granitica* (88.0%). The lowest scavenging effect was observed in *A. orophila*, with only 40.2%. The ranked dose-response DPPH radical scavenging effects was: LW > LG > PW > GB > LA > CH > AO (Figure 1B). A pseudo-IC_50_ for DPPH free radical scavenging and reducing power for each extract was calculated and given in Figure 1C and Table 3, respectively. In the pseudo-IC_50_ assay *L. wooroonooran* (0.21 mg/mL), *L. granitica* (0.22 mg/mL), and *P. willmottii* (0.23 mg/mL) gave better IC_50_ values than the rest of the species which was in accord with the results from the DPPH assay. The pseudo-IC_50_ of *L. wooroonooran* for DPPH free radical scavenging effect was almost equivalent to the standard gallic acid (Figure 1C).

### 2.5. Ferric Reducing Antioxidant Power (FRAP) Activity

The FRAP activity of all seven species at four different concentrations (25 to 500 μg/mL) is summarised in Table 3. FRAP values for each plant were expressed in Fe^2+^ equivalent (mM)/mL of the sample, and ascorbic acid was used as a reference/standard. For all seven tested species, the FRAP value increased with increasing concentration of extracts. *Litsea granitica* showed the highest Fe^2+^ reducing activity at all concentrations (tested concentrations from 25 to 500 μg/mL), with FRAP values ranging from 0.99 ± 0.75 to 8.40 ± 0.54 mM (ascorbic acid being 1.70–29.59 μg/mL), which generated a pseudo-IC_50_ value of 3.19 mg/mL. *Leptospermum wooroonooran* and *P. willmottii* showed similar FRAP activities, with pseudo-IC_50_ values of 4.10 and 4.50 mg/mL, respectively. *Alyxia orophila* had a weak FRAP activity with a pseudo-IC_50_ value of 24.78 mg/mL (Table 3).

### 2.6. Correlation of Pseudo-IC_50_ Values of Antioxidant Activities with TPC and TFC

For total phenolic content, a significant (*p* < 0.007) and positive correlation (R^2^ > 0.7947) was found between TPC and pseudo-IC_50_ values for FRAP, whereas with TFC, a correlation was found (R^2^ > 0.4139), but it was not significant. A weak positive but non-significant correlation was found in the case of pseudo-IC_50_ values for DPPH with TPC and TFC (Table 4).

### 2.7. Cell Viability Assay

**Figure 2:**
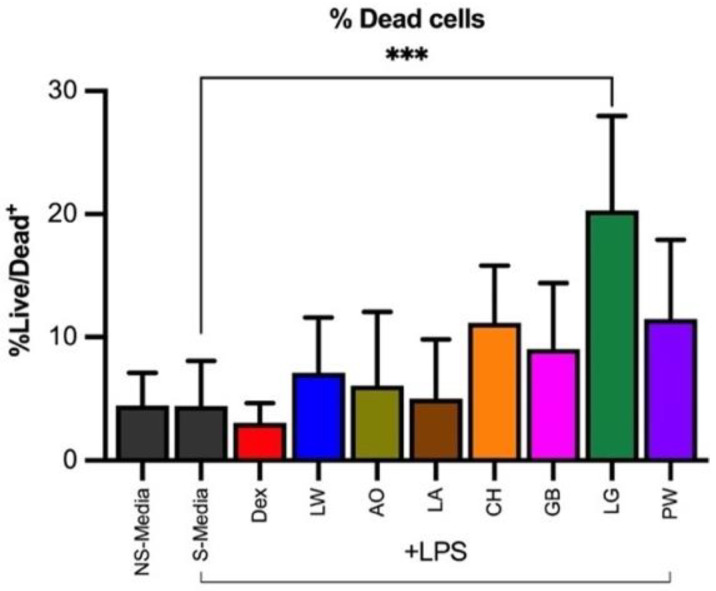
The percentage of dead cells in LPS-stimulated human PBMCs treated with the hydroethanolic extract of seven Wet Tropics plant species in two independent experiments after 20 h incubation. Data expressed as mean ± SD of two independent experiments with blood from two donors (n = 4) (one-way ANOVA was performed in GraphPad prism to find the test significance, where *** *p* < 0.0002. NS-Media—non-stimulated media; S-Media—stimulated media, LW—*Leptospermum wooroonooran*; AO—*Alyxia orophila*; LA—*Linospadix apetiolatus*; CH—*Ceratopetalum hylandii*; GB—*Garcinia brassii*; LG—*Litsea granitica*; PW—*Polyscias willmottii*; Dex—dexamethasone (positive control).

The cell viability of peripheral blood mononuclear cells (PBMCs) co-cultured with hydroethanolic crude extracts (100 μg/mL) from seven plants was determined by staining the cells with a live/dead viability dye (Invitrogen^TM^, eBioscience^TM^ flexible Viability Dye eFluor^TM^ 780, Thermo Fischer Scientific, Scoresby, VIC, Australia), as described in the methods section. The percentage of dead cells was in the range 5.03–20.30% after 20 h incubation. *Litsea granitica* showed the highest percentage (20.30%) of dead cells, which was significant compared to the negative control (Figure 2). The rest of the plant extracts showed a lesser percentage of dead cells (mean < 11% dead cells). Since the tested samples (except *L. granitica*) had a high percentage of cell viability (not significantly different cell death measures when compared with the stimulated control), the PMBCs culture supernatants were further assessed for the presence of inflammatory cytokines released by the cells.

### 2.8. Anti-Inflammatory Activity

**Figure 3:**
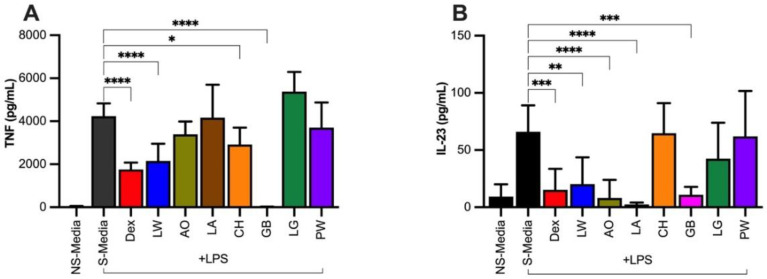
Anti-inflammatory activities of seven Wet Tropics plant extracts at 100 μg/mL. Selected extracts inhibited the production of proinflammatory cytokines in the supernatant of LPS-stimulated PBMCs: (**A**) tumour necrosis factor (TNF); (**B**) interleukin-23 (IL-23). The soluble proinflammatory cytokines were determined in the PBMCs supernatant of HBD (n = 4). PBMCs (1 × 10^6^ cells per well at a final volume of 1 mL) were cultured for 20 h at 37 °C and 5% CO_2_: PBMCs were stimulated with 10 ng/mL LPS. Dexamethasone was used as a positive control. Data provided in mean ± SD (one-way ANOVA was performed in GraphPad prism to test significance, where **** *p* < 0.0001, *** *p* < 0.0002, ** *p* < 0.0021, and * *p* = 0.0332). LW—*Leptospermum wooroonooran*; AO—*Alyxia orophila*; LA—*Linospadix apetiolatus*; CH—*Ceratopetalum hylandii*; GB—*Garcinia brassii*; LG—*Litsea granitica*; PW—*Polyscias willmottii*; Dex—dexamethasone (a positive drug control); NS-Media—non stimulated media; S-Media—stimulated media.

The inhibitory effect of hydroethanolic extracts (100 μg/mL) from seven species on 13 proinflammatory cytokines and chemokines in the supernatant of LPS-stimulated PBMCs was investigated using a Legendplex^TM^ flow assay as described in the methods section. Out of the 13 cytokines and chemokines tested (IL-1β, IFN-α, IFN-γ, TNF, MCP-1, IL-6, IL-8 (CXCL8), IL-10, IL-12p70, IL-17A, IL-18, IL-23, and IL-33), the crude extracts of three species significantly inhibited the production of proinflammatory cytokine TNF (Figure 3A). These three species were *L. wooroonooran* (LW), *C. hylandii* (CH), and *G. brassii* (GB). In addition, *L. wooroonooran* and *G. brassii*, along with *A. orophila* (AO) and *L. apetiolatus* (LA), significantly inhibited the production of IL-23 (Figure 3B). Interestingly, *C. hylandii* extract did not inhibit the production of IL-23. 

## 3. Discussion

Plants are important sources of natural antioxidants. Plant-based natural antioxidants are often sought as health-promoting agents [44,45] as synthetic ones frequently have adverse health effects [46,47]. Many rapid in vitro techniques are used to screen natural products for their antioxidant properties. The DPPH free radical scavenging activity assay, which measures the hydrogen atom donating ability of the sample to the odd electron of a nitrogen atom in DPPH, is one of the most commonly used methods for determining the antioxidant capacity of extracts and molecules [48]. Because there are several potential interferences to this assay [49,50], we also used the antioxidant FRAP assay to confirm the antioxidant capacities of crude extracts of plants that grow in the Wet Tropics of north Queensland, which are predicted to be affected drastically by climate change.

In this study, six out of seven plant extracts tested for their scavenging ability using the DPPH free radical assay showed a high percentage of scavenging activity, with values ranging from 75.4% to 92.3% at the highest tested concentration of 1000 μg/mL. Extracts of *L. wooroonooran*, *L. granitica*, and *P. willmottii* showed the highest percentage of DPPH free radical scavenging activity. The high scavenging activity of these plants could be due to the presence of phenolics and flavonoids identified through qualitative class-based tests, as they contain hydroxyl groups that can transfer hydrogen to the radical centre nitrogen atom in DPPH, thus reducing it to corresponding hydrazine [51,52]. Moreover, the antioxidative mechanism of polyphenols is attributed to their affinity for phospholipid bilayer membranes by forming a hydrogen bond with oxygen atoms on the phospholipid membrane [53].

*Alyxia orophila,* which belongs to the family Apocynaceae, showed the lowest activity in both the DPPH and FRAP antioxidant assays. The low free-radical scavenging and ferric ion reducing activities of *A. orophila* appear to be partially due to the presence of fewer/weaker antioxidant compounds that can donate fewer hydrogen atoms to reduce free radicals (i.e., DPPH and ferric ions in this case).

The Apocynaceae family, including the widely used medicinal plant genus *Catharanthus*, are reported to have low phenolic content [54,55]. For example, studies on *Alyxia* species such as *A.* *reinwardtii* and *A. sinensis* [56,57] have reported comparatively weak antioxidant activities in these species, attributed to lower levels of phenolics and flavonoids in their aerial parts. Iqbal et al. [58] studied the TPC, TFC, and total antioxidant capacity of five plants belonging to the Apocynaceae family, and the highest TPC found was only 60 mg GAE/g tissue (*Catharanthus roseus*), comparable to the highest values found in this study. In addition, *A. orophila* showed the presence of tannins, which can inhibit lipid peroxidation via inhibiting cyclooxygenase enzymes [59] and can chelate ferrous iron (Fe^2+^) that interfere with the Fenton reaction, thereby reducing the formation of hydroxyl radicals from the reaction between Fe^2+^ and hydrogen peroxide [60]. Tannic acid is also known to react effectively in the DPPH assay [61], so further information is needed on potential interferences for this species.

In the FRAP assay, the yellow colour (Fe^3+^-TPTZ complex) in a test sample changes to blue (Fe^2+^-TPTZ) in the presence of reducing antioxidants [62,63]. The FRAP value was expressed in terms of Fe^2+^ equivalent (mM)/mL extract. Using this assay, *L. granitica, L. wooroonooran*, and *P. willmottii* showed the highest FRAP activity, and *A. orophila* the lowest.

Correlation between pseudo-IC_50_ values of antioxidant activities (DPPH and FRAP assays) and TPC and TFC was determined. A significant positive correlation was found between pseudo-IC_50_ values from the FRAP assay and phenolic content (TPC), whereas a weak positive correlation was found between pseudo-IC_50_ values from the DPPH scavenging assay and both TPC and TFC. The strength of the correlation in antioxidant activity with TPC across species may depend on the antioxidant response of different phenolic compounds present in the extracts [64]. In addition, as indicated previously, interference may have come from other chemicals such as sugars and pigments in the crude extracts [65].

Plants growing under stressful conditions (e.g. abiotic stresses), including those in higher altitudes or mountaintop ecosystems [35], are reported to produce more phenolics, flavonoids, and terpenes [22,37,66]. Giupponi et al. (2020) observed the influence of elevation in the secondary metabolites composition of hemp (*Cannabis sativa*) inflorescence, with a higher amount of terpenes in those high-altitude exposed samples compared to their lower altitude counterparts [66]. In the initial phytochemical screening of the plant extracts for their major classes of chemicals, three species (*L. wooroonooran, P. willmottii,* and *L. granitica*) tested positive for terpenoids. In quantitative analysis, these species had high total phenolic and flavonoid content. As shown by qualitative analysis, the presence of 3–4 major classes of polyphenolic compounds in the crude extracts of these three species could be responsible for their high antioxidant activities, as plants containing more diverse groups of phytochemicals are known to give better biological activities [29,39]. When Rana et al. (2020) evaluated the major classes of phytochemical compounds and antioxidant activities in the wild population of medicinal species *Coleus forskohlii* collected from five different locations (600–2000 masl), there was a significant (*p* < 0.05) increase in the total phenolic, flavonoid and terpenoid content with increasing altitude, which resulted in higher antioxidant activities, including DPPH scavenging and FRAP activity [67].

Plants showing strong antioxidant activities are expected to possess better anti-inflammatory properties. This study further tested hydroethanolic crude extracts from seven plant species for their anti-inflammatory potential in LPS-stimulated human PBMCs assay. Three species—*G. brassii, C. hylandii*, and *L. wooroonooran*—showed significant inhibition of TNF. Tumor necrosis factor is a proinflammatory cytokine produced by immune cells, including M1 macrophages or monocytes, and is upregulated in most inflammatory conditions and induces apoptosis and inflammation [68]. The production of TNF increases in the presence of ROS, which accentuates oxidative stress at inflammation sites, and indirectly causes fever [69,70]. *Leptospermum wooroonooran* and *G. brassii*, along with *A. orophila* and *L. apetiolatus,* also significantly inhibited IL-23. Interleukin IL-23 is also proinflammatory and has an indirect but vital role in causing inflammation. It initiates the differentiation of Th17 cells in proinflammatory situations in the presence of transforming growth factor-beta (TGF-β) and IL-6. Activated Th17 cells, in turn, secrete a series of other proinflammatory cytokines, including TNF and IL-17 [71]. Enhanced levels of IL-23 were reported in the skin of psoriasis patients [72], in the bowel wall of chronic inflammatory bowel disease patients [73] and in the synovial membrane tissues of rheumatoid arthritis patients [74].

The seven plant species were tested for toxicity and all except for *L. granitica* (which caused 20.3% cell death in cell viability assay) showed low toxicity, with less than 11% cell death after 20 h. A lower toxicity profile and potent antioxidant and anti-inflammatory activities of crude extracts merits more in-depth phytochemical and pharmacological studies of *G. brassii* and *L. wooroonooran*. In concert with the pseudo-IC_50_ correlations, the strongest prospects for new anti-inflammatory lead compounds would be in the phenolic class of compounds in extracts from *L. wooroonooran, G. brassii,* and *C. hylandii* (noting the latter had a low recovery).

## 4. Materials and Methods

### 4.1. Plant Collection and Reagents

**Figure 4:**
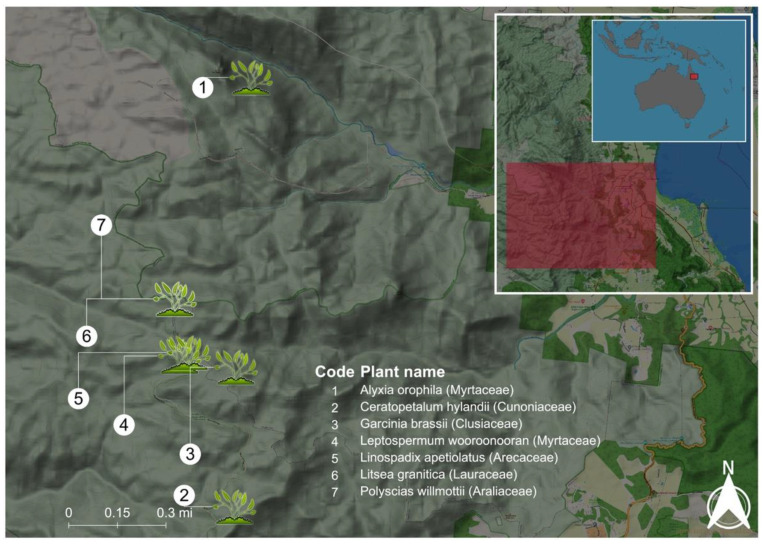
A map showing the collection sites of seven Wet Tropics’ mountaintop plants studied here. GPS coordinates for: 1 (latitude: 16.448505879; longitude: 145.284402623); 2 (latitude: 16.548323; longitude: 145.280569); 3 (latitude: 16.5158965385; longitude: 145.2809076538); 4 (latitude: 16.5133000000; longitude: 145.2678000000); 5 (latitude: 16.5125000000; longitude: 145.2700000000); 6 (latitude: 16.5000000000; longitude: 145.2667000000); 7 (latitude: 16.5000000000; longitude: 145.2667000000). Plant coordinates were obtained from our collection authority/permit no. BCA20-002698. The map scale is in miles (mi).

Aerial parts (leaves and twigs) of seven mountaintop plant species were collected (collection authority/permit no. BCA20-002698) from Mount Lewis National Park, Queensland, Australia (Figure 4), at 1000–1200 m above sea level in July 2020. The samples were identified by a senior taxonomist (co-author D.C.) and vouchered at the Australian Tropical Herbarium, James Cook University, Smithfield, Australia (herbarium code CNS).

Fresh aerial parts were washed and then dried in an oven. Dried samples were packed in sealed paper bags and stored in a cold room at 4 °C until use. All chemicals used in this study were purchased from either Sigma-Aldrich (Melbourne, VIC, Australia) (Folin-Ciocalteu reagent, gallic acid, ethanol, sodium carbonate, aluminium chloride, ferric chloride, ferric sulphate, L-ascorbic acid, 2,4,6-tripyridyl-S-triazine (TPTZ), 2,2-diphenyl-1-picrylhydrazyl (DPPH), quercetin, glacial acetic acid, and hydrochloric acid) or ChemSupply (Gillman, SA, Australia) (methanol and ethanol).

### 4.2. Extract Preparation

Plant extracts were prepared using the previously described method [41,43,75,76]. Briefly, for each sample, 25 grams (g) of the dried coarse powdered sample (ground using a NutriBullet) were extracted with 150 mL 80% ethanol and left for 2 h (constantly stirring). The solvent was replaced every 2 h (a total of three rounds of extraction). The combined extracts were filtered using a Sterricup quick vacuum-driven disposable filtration system (0.22 μm PES, 500 mL, Merck, Kenilworth, NJ, USA). The solvent was evaporated using a rotary evaporator (Heidolph, Germany). The percentage yield of extract was calculated using the formula:

$$\%\;Yield=\frac{mass\;of\;the\;extract}{mass\;of\;plant\;material}\times \frac{100}{1}$$

### 4.3. Qualitative Screening for Major Classes of Phytochemicals

Qualitative phytochemical screening for the presence of alkaloids, flavonoids, steroids, terpenoids, tannins, saponins, and glycosides was performed using methods from Iqbal et al. (2015) [77], Shah and Hossain (2014) [78], and Wangchuk et al. (2011) [41]. Briefly, 350 mg of crude extract of each sample was dissolved in 7 mL of 80% ethanol and divided into seven parts (7 parts × 1 mL) and conducted the following seven tests:

1. Alkaloid test: Dragendorf’s reagent (1 mL) was added to the sample, and the formation of orange precipitate confirmed the presence of alkaloid.
2. Steroid test: chloroform (2 mL) was added to the sample, followed by a few drops of acetic anhydride. Next, the reaction mixture was boiled in a water bath and cooled in the ice water. Concentrated sulphuric acid (1 mL) was then added, and the formation of a brown ring at the junction and change of the upper layer into green confirmed the presence of steroid.
3. Terpenoid test: chloroform (2 mL) was added to the sample, followed by a few drops of concentrated sulphuric acid, and the formation of reddish-brown coloration at the interface confirmed the presence of terpenoid.
4. Tannin test: a few drops of 10% aqueous ferric chloride was added to the sample, and the formation of a black precipitate or blue-green coloration confirmed the presence of tannin.
5. Saponin test: distilled water was added and the sample was shaken for few minutes. The presence of foam/frothing that persisted after warming in the water bath (37 °C) for 5 min confirmed the presence of saponin.
6. Anthraquinone glycoside test: 5% sulphuric acid (1 mL) and chloroform (2 mL) were added to the sample, followed by shaking the lower layer with dilute ammonia. Rose pink to red colour of the ammoniacal layer confirmed a positive test result.
7. Cardiac glycoside test: glacial acetic acid (2 mL) was added to the sample, followed by a few drops of 10% ferric chloride and then 5% sulphuric acid (1 mL). The formation of a brown ring at the interface confirmed the presence of cardiac glycoside.

### 4.4. Quantitative Phytochemical Estimation

#### 4.4.1. Total Phenolics Content (TPC)

TPC was determined following a modified method based on Djeridane et al. (2006) [79] and Singleton et al. (1974) [80]. Briefly, 100 μL of extract (1 mg/mL) was added to 500 μL Folin-Ciocalteu reagent (1N) and incubated at room temperature for 3 min. After 3 min, 1 mL of 20% Na_2_CO_3_ solution was added. The final reaction mixture was shaken and left in the dark for 2 h at room temperature. After 2 h incubation, a 250 μL aliquot was removed and placed in one well of a 96-well flat-bottom plate (Falcon^®^, Corning, NJ, USA); absorbance was measured at 760 nm using a multifunctional microplate reader (SPECTROstar^®^ Omega, Ortenberg, Germany). The reader had previously been calibrated at this wavelength using a calibration curve of gallic acid (0 to 1500 μg/mL). The total phenolic content was expressed in milligrams (mg) gallic acid equivalent (GAE) per g dry extract.

#### 4.4.2. Total Flavonoids Content (TFC)

TFC was determined following a method by Djeridane et al. (2006) and Quettier-Deleu et al. (2000) [79,81] based on the formation of a coloured flavonoid–aluminium complex with maximum absorption at 430 nm. Briefly, in a 5 mL test tube, 1 mL of extract (1 mg/mL) and 1 mL of 2% AlCl_3_.6H_2_O ethanolic solution were mixed well and left at room temperature for 15 min. After this, the absorbance was measured at 430 nm using the microplate reader (SPECTROstar^®^ Omega, BMG Labtech, Mornington, VIC, Australia). A standard curve was prepared on the reader using a quercetin solution (0 to 200 μg/mL). The TFC was expressed as mg of quercetin equivalents (QE) per g of dry extract for each extracted sample.

### 4.5. Antioxidant Activity Assays

#### 4.5.1. 2,2-Diphenyl-1-picrylhydrazyl (DPPH) Assay

The DPPH free radical scavenging activity of each hydroethanolic crude extract from seven plant species was evaluated using a method described by Brand-Williams et al. [42] and Yeshi et al. [43]. Briefly, 100 μL of sample extract (25, 100, 250, 500, 1000 μg/mL) or 100 μL of the positive control (gallic acid, at the same concentrations) were mixed with 150 uL of an ethanol solution of DPPH (0.1 mM) and placed in a 96-well flat-bottom plate (Falcon^®^, Corning, NJ, USA). The reaction mixture was shaken and left in the dark at room temperature for 15 min. The absorbance was then measured at 517 nm in the microplate reader. All sample extracts and controls were evaluated in triplicate. The percentage of DPPH free radical scavenged was calculated using the equation below:

$$\%\;DPPH\;free\;radical\;scavenging\;activity=\left(\frac{ODc-ODs}{ODc}\;\right)\;\times \;100$$

where *OD_C_* is the absorbance value of the control, and *OD_S_* is the absorbance value of the tested sample or positive control.

#### 4.5.2. Ferric Reducing Antioxidant Power (FRAP) Assay

The ferric reducing antioxidant power or FRAP activity of all crude extracts was determined using the method described by Benzie and Strain [62]. Briefly, 10 μL of the sample or positive control (L-Ascorbic acid) (25, 100, 250, 500 μg/mL) was added to 300 μL of freshly prepared FRAP reagent (20 mM FeCl_3_·6H_2_O solution, 10 mM TPTZ solution in 40 mM HCl and 300 mM acetate buffer in a ratio 1:1:10, *v*/*v*/*v*) in a 96-well flat-bottom plate (Falcon^®^, Corning, NY, USA). The reaction mixture was left for 15 min in an incubator (37 °C). After 15 min, samples were taken out of the incubator and left at room temperature for 5 min. Subsequently, their absorbance was measured at 593 nm with a microplate reader. All extracts and controls were evaluated in triplicate. A calibration curve was measured on the plate reader using ferrous sulphate (FeSO_4_·7H_2_O, 0–2000 μg/mL), and the FRAP data were expressed in terms of equivalent mM of Fe^2+^/mL of dry extract.

### 4.6. Calculation of Pseudo-IC_50_ of Antioxidant Activities

A pseudo-IC_50_ of antioxidant activities for each extract was calculated according to the following procedure: Scavenging/reducing ratios (y) were plotted against the sample concentrations (x) at all five points, and the respective regression line (y = ax + b) was generated. The pseudo-IC_50_ was calculated from the equation (y = ax + b) by substituting ‘y’ with 50 and ‘x’ with the extract concentration from the fit. This procedure was used for both the DPPH and FRAP assays.

### 4.7. In Vitro Anti-Inflammatory Activity and Quantification

#### 4.7.1. PBMCs Culture and Sample Treatment

Ethics for this assay was approved by the James Cook University Human Research Ethics Committee (approval number H8523). Peripheral blood mononuclear cells (PBMCs) were separated from the venous blood of two healthy blood donors (HBD) supplied by the Australian Red Cross Lifeblood, Australia, using the Ficoll-Paque PLUS (GE Health) density gradient method from STEMCELL technologies^TM^. They were cryopreserved in filtered heat-inactivated Fetal Bovine Serum (FBS) (Corning #35-076-CV) containing 10% dimethyl sulfoxide (DMSO, Sigma-Aldrich). PBMCs were then advanced to the immune assay. Briefly, PBMCs were stimulated with 10 ng/mL lipopolysaccharide (LPS) (Sigma-Aldrich). For all tests, 1 × 10^6^ cells in 100 μL of R-10 media (RPMI-1640 (Gibco), containing 10% heat-inactivated FBS, 100 U/mL Penicillin, and 100 μg/mL Streptomycin (Gibco) were seeded into the wells of 96-well U-bottom culture plates (Falcon^®^, Corning, NY, USA). Stimulated PBMCs were treated in quadruplets individually with the hydroethanolic crude extracts of selected plants (100 μg/mL in cell culture media with 0.5% DMSO). PBMCs culture treated with 100 μg/mL of dexamethasone was used as a positive control, and R-10 media with DMSO was the negative control. Culture plates were incubated for 20 h at 37 °C in a 5% CO2 incubator. Following overnight incubation, plates were centrifuged (277× *g* force, 4 °C for 5 min), and the culture supernatants were collected for cytokine analysis.

#### 4.7.2. Determination of Cell Viability

Prior to testing for anti-inflammatory activity, a cell viability assay was performed on PBMCs by co-culturing them with hydroethanolic crude extracts from seven plants to determine their effect on cell health (cytotoxicity) [82]. After collecting the supernatant, cells were stained with live/dead viability dye (LIVE/DEAD^TM^ fixable near-IR Dead Cell Stain Kit, for 633 or 635 nm excitation, cat. No. L34975) following the manufacturer’s manual. After 30 min, stained cells were washed with 200 μL PBS 2%FBS twice. Cells were finally resuspended in 100 μL 2% paraformaldehyde (PFA)/PBS buffer and analysed using a custom LSRFortessa X20 (BD Biosciences). The live/dead viability dye binds amines and gives dim staining in the case of live cells. In contrast, the dye penetrates damaged membranes of dead cells, binding to interior and exterior amines, giving brighter/intense staining, thus enabling the identification of viable cell populations. The percentage of live/dead cells was determined by analysing flow cytometry data (in FCS format) using FlowJo 10.8.1 version software (Ashland, OR, USA). Before determining the %Live/Dead dye positive cells, doublets were excluded from the analysis by gating FSC-H (forward scattering height) versus FSC-A (forward scattering area). Based on cell viability results, PBMCs culture supernatants were further analysed for cytokine stimulation/inhibition by sample treatments.

#### 4.7.3. Quantification of Proinflammatory Cytokines

For cytokine profiling of the PBMCs culture supernatants, a Legendplex^TM^ assay was conducted using a customised Legendplex^TM^ multi-analyte flow assay kit (Cat. No. 740808, lot no. B291817) for detection of 13 human inflammatory cytokines purchased from BioLegend^®^, USA. Levels of all 13 cytokines (interleukin IL-1β, interferon alpha (IFN-α), IFN-γ, tumor necrosis factor (TNF), monocyte chemoattractant protein-1 (MCP-1), IL-6, IL-8 (CXCL8), IL-10, IL-12p70, IL-17A, IL-18, IL-23, and IL-33) were quantified according to the manufacturers’ instructions, analysed on an LSRFortessa X20 (BD Biosciences). For the determination of cytokine concentrations (all defined as pg/mL), flow cytometry data files were analysed in a cloud-based software platform from the BioLegend^®^ website (San Diego, CA, USA). All tests were performed in quadruplets and are expressed as mean ± standard deviation (SD).

### 4.8. Statistical Analysis

Antioxidant and anti-inflammatory data were expressed as the mean ± SD from two independent experiments. Data were further analysed by a one-way (ANOVA) in GraphPad Prism version 8.4.3 (San Diego, CA, USA), and the group means were compared using Dunnett’s Multiple Range Test. A probability of *p* < 0.05 was considered significant.

## 5. Conclusions

Of the seven plant species studied, hydroethanolic extracts from six species showed moderate-to-strong antioxidant activities (free radical scavenging and reducing power) in a dose-dependent manner. There was a positive correlation between pseudo-IC_50_ values of ferric ion reducing antioxidant power (FRAP) and total phenolic content (TPC). The seven plants’ hydroethanolic extracts also showed varying inhibitory effects on two proinflammatory cytokines (TNF and IL-23). *Leptospermum wooroonooran* and *G. brassii* showed significant inhibitory effects on both TNF and IL-23 in LPS-stimulated human PBMCs. The results from the current study form a scientific basis for further investigation of mountaintop plants as a source of antioxidant agents and novel anti-inflammatory chemical leads in biodiscovery. Considerable antioxidant activity and significant inhibitory effects of extracts on proinflammatory cytokines warrant further studies on at least four (*G. brassii, L. wooroonooran, L. apetiolatus,* and *C. hylandii)* out of seven species of mountaintop plants investigated in this study.

## Figures and Tables

**Table 1:** Voucher specimen numbers of the seven Wet Tropics plants and their percentage yield of crude extracts.

Plants (Family)	Specimen Voucher Number	Abbreviation	Extract Yield (%)
*Alyxia orophila* Domin (Myrtaceae)	D.M. Crayn 1168 (CNS 135636.1)	AO	9.5
*Ceratopetalum hylandii* Rozefelds & R.W.Barnes (Cunoniaceae)	S.J. Worboys 1682 (CNS 148538.1)	CH	3.5
*Garcinia brassii* C.T. White (Clusiaceae)	S.J. Worboys 1681 (CNS 148513.2)	GB	8.2
*Leptospermum wooroonooran* F.M.Bailey (Myrtaceae)	D.M. Crayn 1172 (CNS 135635.1)	LW	16.8
*Linospadix apetiolatus* Dowe & A.K.Irvine (Arecaceae)	D.M. Crayn 1572 (CNS 145626.1)	LA	5.7
*Litsea granitica* B.Hyland (Lauraceae)	D.M. Crayn 1437 (CNS144467.1)	LG	10.4
*Polyscias willmottii* (F.Muell.) Philipson (Araliaceae)	D.M. Crayn 1575 (CNS 145629.2)	PW	16.4

**Table 2:** Wet Tropics’ plant extracts showing positive tests for major classes of phytochemicals.

Plants	Alkaloid	Steroid	Terpenoid	Tannin	Saponin	Anthraquinone Glycoside	Cardiac Glycoside
*Alyxia orophila*	−	++	+	+++	+++	−	+
*Ceratopetalum hylandii*	+++	++	+++	+++	−	−	+++
*Garcinia brassii*	+	+	−	+++	+++	+	−
*Leptospermum wooroonooran*	−	+	+	+++	−	−	−
*Linospadix apetiolatus*	−	−	−	+++	++	−	+
*Litsea granitica*	−	++	+++	+++	+	−	−
*Polyscias willmottii*	+	++	+++	++	−	−	−

+++ = high/abundant, ++ = moderate, + = trace/weak, − = absent.

**Table 3:** Ferric reducing antioxidant power (FRAP) values of selected seven Wet Tropics species.

Plants	Extract Concentration				Pseudo-IC_50_ (mg/mL)
	25 μg/mL	100 μg/mL	250 μg/mL	500 μg/mL	
*Alyxia orophila*	0.26 ± 0.20	0.91 ± 0.71	0.93 ± 0.72	1.39 ± 0.23	24.78
*Ceratopetalum hylandii*	0.48 ± 0.42	0.67 ± 0.13	1.78 ± 0.74	2.85 ± 0.45	9.56
*Garcinia brassii*	0.27 ± 0.07	1.09 ± 0.38	2.02 ± 1.04	3.23 ± 0.23	8.28
*Leptospermum wooroonooran*	0.63 ± 0.07	1.57 ± 0.07	3.48 ± 0.33	6.39 ± 0.25	4.10
*Linospadix apetiolatus*	0.65 ± 0.47	0.97 ± 0.53	0.94 ± 0.07	1.90 ± 0.33	20.59
*Litsea granitica*	0.99 ± 0.75	2.50 ± 1.00	5.10 ± 0.74	8.40 ± 0.54	3.19
*Polyscias willmottii*	0.66 ± 0.29	1.44 ± 0.33	3.60 ± 0.35	5.80 ± 0.66	4.50
Ascorbic acid	1.70 ± 0.25	6.37 ± 0.75	14.59 ± 1.95	29.59 ± 0.51	0.85

FRAP values are expressed as mean ± standard deviation (n = 3). Ascorbic acid is used as standard compound. Pseudo-IC_50_ is the half maximal concentration of the sample crude extract that is required for 50% reduction of ferric-tripyridyl triazine (Fe^3+^-TPTZ) complex to ferrous-tripyridyl triazine (Fe^2+^-TPTZ).

**Table 4:** Correlations between the pseudo-IC_50_ values of antioxidant activities and phenolics and flavonoids content of samples tested.

Assays	Correlation R^2^	
	Phenolics (TPC)	Flavonoid (TFC)
IC_50_ of DPPH radical scavenging ability	0.4933	0.1698
IC_50_ of FRAP activity	0.7947 **	0.4139

** indicates significance at *p* = 0.007.

## Data Availability

Data is contained within the article.
